# Accuracy of polyether and vinylpolysiloxane impressions when using different types of 3D-printed impression trays - an in vitro study

**DOI:** 10.1007/s00784-024-05962-2

**Published:** 2024-09-30

**Authors:** Stefan Rues, David Depré, Thomas Stober, Peter Rammelsberg, Andreas Zenthöfer

**Affiliations:** Department of Prosthodontics, University of Heidelberg, Im Neuenheimer Feld 400, 69120 Heidelberg, Germany

**Keywords:** Open implant impression, Accuracy, 3D-printed trays, Polyether, Polyvinylsiloxane, Peel bond strength

## Abstract

**Objectives:**

To investigate dimensional accuracy of polyether (PE) and vinylpolysiloxane (VPS) impressions taken with manually fabricated and 3D-printed trays.

**Materials and methods:**

To evaluate impression accuracy, highly precise digital data of a metallic lower jaw model with prepared teeth (regions 34 and 36), an implant (region 47) and three precision balls placed occlusally along the dental arch served as reference. PE (Impregum, 3M Oral Care) and VPS (Aquasil, Dentsply Sirona) impressions (*n* = 10/group) were taken with trays fabricated using different materials and manufacturing techniques (FDM: filament deposition modeling, material: Arfona Tray, Arfona; printer: Pro2, Raise3D; DLP: digital light processing, material: V-Print Tray, VOCO, printer: Max, Asiga; MPR: manual processing with light-curing plates, material: LC Tray, Müller-Omicron) including an open implant impression. Scans of resulting stone models were compared with the reference situation. Global distance and angular deviations as well as local trueness and precision for abutment teeth and scan abutment were computed. Possible statistical effects were analyzed using ANOVA.

**Results:**

Clinically acceptable global accuracy was found (all mean absolute distance changes < 100 μm) and local accuracy for single abutments was excellent. All factors (abutment type, impression material, tray material) affected global accuracy (*p* < 0.05). In particular with PE impressions, MPR trays led to the best accuracies, both in horizontal and vertical direction.

**Conclusions:**

Within the limitations of this in vitro study, impression accuracy was high in use of both polyether and vinylpolysiloxane combined with different 3D-printed and customized trays making them recommendable for at least impressions for smaller fixed dental prostheses. Manually fabricated trays were overall still the best choice if utmost precision is required.

**Clinical relevance:**

Based on the results of this study, use of innovative CAD-CAM fabrication of individual impression trays fulfills the perquisites to be a viable option for impression making. In the sense of translational research, performance should be proved in a clinical setting.

## Introduction

In prosthetic dentistry the transfer of the clinical situation in terms of fabrication of dental protheses by means of impression making is a key step [1]. Besides the use of intraoral scanners (IOS), polyether (PE) and vinylpolysiloxane (VPS) impression materials combined with prefabricated trays are still widely common [1, 2]. Digital impression making by use of IOS, however, saves waste regarding used impression and tray materials, allows correction of local impression imperfections and is preferred by the patients because of comfort. Whilst small clinical situation for small restorations such as single crowns and short-span fixed dental prostheses (FDPs) can be equivalently or even better assessed using IOS [3, 4], conventional impressions are still outperforming IOS with regard to impression accuracy when restorations with more than 4 units are to be fabricated [4]. In addition, for larger restorations the adjustment of the dynamic occlusion is of great importance, more adequately possible with physical stone models [4]. Thus, clinicians have still to fall back to conventional VPS and PE impressions when it comes to more complex and long-span restorations. Both material groups have their advantages and disadvantages, to name a few, VPS offers a relatively neutral taste and cured impressions are storable for long periods of time; PE includes initially hydrophilic behavior and should be therefore be present with an advantaged flow on tooth structures [5, 6]. For the sake of completeness, one should mention that there are also hybrid impression materials which aim to combine the favorable properties of both material groups [7] and, in addition, quick-setting derivates for smaller restorations [8, 9]. Oral hygiene and periodontal health and therefore bleeding tendency, the location of the preparation margin, gingiva retraction, saliva and the angulation / under-cuts of the abutment tooth, amongst others, all affect the accuracy of impressions, too [10–12]. The same is true for post-procedural measures like disinfection [13, 14]. Moreover, differences in the resulting accuracy were also seen in dependence of the impression technique used [15]. A major impact is also attributed to the type and design of the impression trays [16–22]. For final impressions for FDPs, metal trays with flanged margins (border-lock) are recommended as they offer torsional rigidity and inherent stability [17, 22]. The border-lock design combined with the use of tray adhesives seems to hinder detachment of the cured impression resulting in detorsions. Nonetheless, validated hygiene processes enhance the increased use of single-use plastic trays, away from the gold standard of the metal variant as cleaning and sterilization is time- and cost-consuming. The resulting waste, however, is another concern. On the other hand, for some indications such as the pick-up impression of implants, individual / customized trays are, anyway, essential or more easily to adjust. A previous study investigated the dimensional accuracy of prefabricated plastic trays for impression making for tooth-supported fixed dental prostheses and found no clinically relevant differences compared with the use of metal trays, especially for 3-unit bridges or single crown the difference was marginal [23].

CAD-CAM technology in addition is going forward for various dental applications including fabrication of impression trays. 3D-printed resin and filament trays becoming more and more popular [24, 25]. A previous study found that 3D-printed trays led to a higher impression accuracy compared to individual hand-made resin trays with implant impression taking [21]. A further study confirmed this outcome for impressions of the edentulous jaws. Another study postulated that the hand-made trays led to best possible accuracy [20]. Especially filament printed tray materials become maybe biodegradable in the future could combine positive properties in reduction of plastic waste and cleaning and sterilizations costs. However, according to literature research of the authors, no information is available about the accuracy of those impressions.

Therefore, the aim of this laboratory study conducted in 2023 was to investigate the local and global accuracy of three types of customized impression trays (printed resin tray, printed filament tray and individually fabricated tray by use of plate resin) each used with VPS and PE in single-time two phase technique for different fixed dental prosthesis situations. The study hypothesis was that impressions’ accuracy was significantly affected by selection of impression as well as by tray material.

## Materials and methods

### General material properties and peel bond strength (pre-test)

In this in-vitro study, three different tray materials coming along with different manufacturing technologies were investigated:

- Test group “FDM”,
  Arfona Tray (Weithas, Lütjenburg, Germany, LOT 08272001), processed by fused deposition modeling (FDM; Pro2, Raise; Rotterdam, The Netherlands). Objects were printed at 235°C temperature, a nozzle diameter of 0.4 mm and a layer thickness of 0.2 mm. Objects were printed with full density, i.e., there was no inner filling with reduced density.
- Test group “DLP”,
  V-Print Tray (VOCO, Cuxhaven, Germany, LOT 2044132), processed by digital light processing (DLP; Max, Asiga, Erfurt, Germany) with 62 μm pixel size. Printing parameters were given by the respective material bibliography provided by the manufacturer and objects were printed with 0.1 mm layer thickness, Postprocessing ended with final light-curing (2 × 2000 flashes, Otoflash G171, NK-Optik, Baierbrunn, Germany).
- Test group “MPR”,
  LC Tray (Müller-Omicron, Lindlar, Germany, LOT 227538), manual processing of prefabricated resin plates with 2.4 mm thickness and final light curing from each side for 5 min (Omnilight Tray cure-unit, Omnident GmbH, Rodgau Nieder-Roden, Germany).

In a first pre-test, beams with h = 3 mm (FDM and DLP, the maximum wall thickness in the CAD software during tray design is 3 mm) or h = 2.4 mm thickness (MPR, thickness of the prefabricated resin plates) were tested. The other beam dimensions, i.e., b = 10 mm width and L = 64 mm length, were chosen according to ISO 20795. One half of the specimens was loaded immersed in water at 37°C, the other half under dry conditions at room temperature in a 3-point bending test with a span of 50 mm and a cross-head speed of 5 mm/min. For each beam (*n* = 10 / subgroup differing in material and testing condition), Young´s modulus and bending strength were determined.

**Fig. 1:**
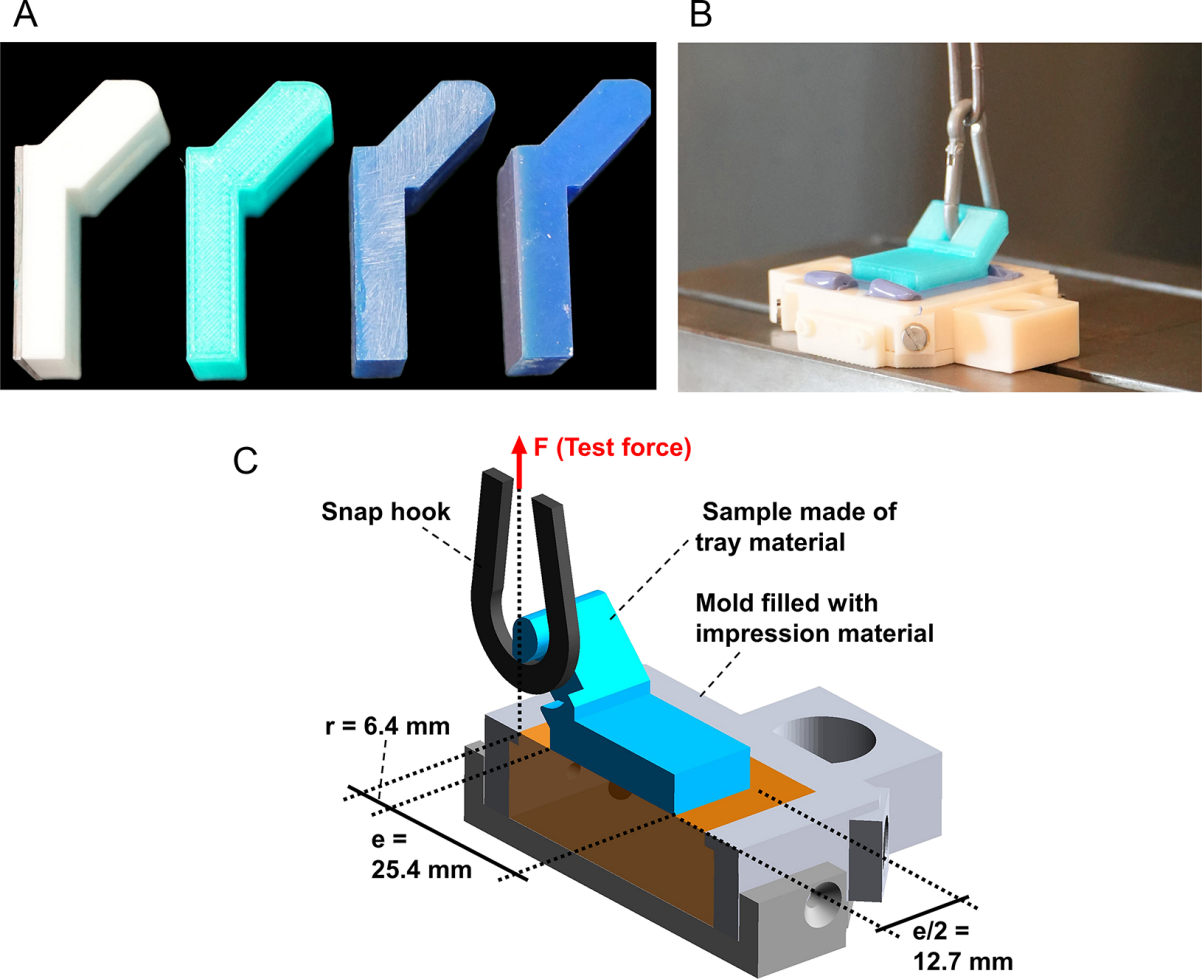
Samples for the peel-bond-strength test (**a**). From left to right: sample complemented with a stainless steel plate on the lower side (Control), FDM sample, DLP sample, and sample complemented with light curing resin on the lower side (MPR). Samples were debonded with a tensile test (**b**). All relevant dimensions can be seen in the depicted scheme (**c**)

In a second step of the pre-test peel bond strength was evaluated. When making impressions – especially with custom-made trays without border-lock - a concern is detaching of impression material from the tray surface. Therefore, in a second pilot study, peel bond strength values between specimens made of the different tray materials as well as stainless steel as control and the two different low-viscosity impression materials (PE: polyether, Impregum Penta H Duo Soft, LOT 9921089, 3M Oral Care, Seefeld, Germany and VPS: vinylpolysiloxane, Aquasil Ultra+ Heavy Regular Set, Dentsply Sirona, Charlotte, NC, USA) were investigated (*n* = 12 per subgroup differing in tray material and impression material). The setup for a peel bond strength test was already published by another research group [26]: A test specimen (Fig. 1a) with quadratic base area (e = 25.4 mm edge length) and a handle providing a lever arm of *r* = 6.4 mm with respect to the nearest edge of the base area was placed on a mold filled with impression material after the base area was coated with the respective tray adhesive (PE: Polyether Contact Tray Adhesive, LOT 9141163, 3M Oral Care, VPS: Panasil Haftlack, LOT ND210081, Kettenbach, Eschenburg, Germany). After a setting time of 15 min, debonding tests were carried out in a universal testing device (Z005, Zwick/Roell, Ulm, Germany) by lifting the handle upwards with 10 mm/min (cf. Figure 1b and c). Peel bond strength values were calculated by dividing the maximum recorded force by the edge length e of the specimen.

### Accuracy of open impressions with individual trays

#### Reference model and reference data

An already existing reference model consisting of a steel base resembling a lower jaw and CoCr teeth welded to the surface was used in this investigation. Prepared abutment teeth 34 and 36 simulated the clinical case of a 3-unit fixed partial denture. In addition, three stainless steel precision balls (3.175 mm in diameter, quality G3) were welded to the occlusal surface in regions 37 (PB_1_), 46 (PB_2_), and between 11 and 21 (PB_3_). The existing model was modified by placement of a bone-level implant (Standard plus, 4.1 mm diameter, Straumann) in region 47. After removal of composite resin (applied in previous studies) from the undercut regions of the precision balls, coordinate measurement was performed (MarVision MS 222, Hexagon Metrology, Stockholm, Sweden, about 200 measurement points for each element, accuracy < 2 μm) for the abutment teeth, the scan body (not the impression post, since the scan body indicates the implant position and orientation as later with the stone models) screwed to the implant, and the precision balls to determine their spatial position. Highly precise individual surface data with high resolution already existed for abutment teeth 34 and 36. This data with a 50 μm point grid and < 1 μm accuracy had been assessed by the NanoFocus company (NanoFocus, Oberhausen, Germany) with µscan custom and CF 4 sensor before the abutment teeth had been welded to the model base. For the scan body, no additional data was obtained but the reference surface data provided by the manufacturer in a commercial dental design software (Dental Designer Version 2020, 3shape) included in the reference data set. At the end, individual data with high resolution was aligned with the coordinate measurement of the whole model. With the center points ($\:{\text{P}}_{1}, {\text{P}}_{2}, {\text{P}}_{3}$) of the precision balls (PB_1_, PB_2_, PB_3_), a global coordinate system was defined:

- Origin located at $\:{\text{P}}_{1}$
- x-axis directed along the vector$\:\overrightarrow{{\text{P}}_{1}{\text{P}}_{2}}$
- xy-plane defined by the points $\:{\text{P}}_{1}$, $\:{\text{P}}_{2}$, $\:{\text{P}}_{3}$
- y-axis oriented in anterior direction.

Reference points were also defined for the abutment teeth and the implant abutment (points $\:M,P,I$) at the respective finishing line center points. The axes of the abutment teeth ($\:{\overrightarrow{a}}_{M}$, $\:{\overrightarrow{a}}_{P}$ were chosen parallel to the z-axis of the global coordinate system and the orientation of the scan body´s axis was identical to the implant axis (which deviated slightly from the z-axis). To enable evaluation of inaccuracies in horizontal and vertical direction separate from each other, a projection plane parallel to the xy-plane was defined. After projection of all reference points into this plane along the z-axis (e.g., $\:{\text{P}}_{1}\to\:{\text{P}}_{1}^{{\prime\:}}$), all 15 horizontal reference distances defined by $\:{\text{P}}_{1}^{{\prime\:}}$_,_$\:{\text{P}}_{2}^{{\prime\:}},{\text{P}}_{3}^{{\prime\:}},\:{M}^{{\prime\:}},{P}^{{\prime\:}},{I}^{{\prime\:}}\:$were calculated (Table 1). In the vertical direction, z-axis differences between the reference points were of interest. Per definition of the coordinate system (both for reference and later scan data), z-coordinates of all precision balls centers were zero. Thus, vertical deviations were only evaluated between the plane $\:{\text{P}}_{1/2/3}$ and all abutment tooth centers as well as between all abutment tooth center pairs.

**Table 1:** Horizontal reference distances, sorted in ascending order

Distance between		Distance [mm]
$\:{\text{P}}_{1}^{{\prime\:}}$	$\:{\text{M}}^{{\prime\:}}$	8.621
$\:{\text{P}}_{2}^{{\prime\:}}$	$\:{\text{I}}^{{\prime\:}}$	9.796
$\:{\text{M}}^{{\prime\:}}$	$\:{\text{P}}^{{\prime\:}}$	16.047
$\:{\text{P}}^{{\prime\:}}$	$\:{\text{P}}_{3}^{{\prime\:}}$	18.040
$\:{\text{P}}_{1}^{{\prime\:}}$	$\:{\text{P}}^{{\prime\:}}$	23.613
$\:{\text{M}}^{{\prime\:}}$	$\:{\text{P}}_{3}^{{\prime\:}}$	30.993
$\:{\text{P}}_{3}^{{\prime\:}}$	$\:{\text{P}}_{2}^{{\prime\:}}$	31.922
$\:{\text{P}}_{1}^{{\prime\:}}$	$\:{\text{P}}_{3}^{{\prime\:}}$	35.914
$\:{\text{P}}_{1}^{{\prime\:}}$	$\:{\text{P}}_{2}^{{\prime\:}}$	40.330
$\:{\text{P}}^{{\prime\:}}$	$\:{\text{P}}_{2}^{{\prime\:}}$	41.230
$\:{\text{P}}_{3}^{{\prime\:}}$	$\:{\text{I}}^{{\prime\:}}$	41.712
$\:{\text{M}}^{{\prime\:}}$	$\:{\text{P}}_{2}^{{\prime\:}}$	42.456
$\:{\text{P}}_{1}^{{\prime\:}}$	$\:{\text{I}}^{{\prime\:}}$	46.527
$\:{\text{M}}^{{\prime\:}}$	$\:{\text{I}}^{{\prime\:}}$	49.930
$\:{\text{P}}^{{\prime\:}}$	$\:{\text{I}}^{{\prime\:}}$	50.429

#### Design and fabrication of individual trays

**Fig. 2:**
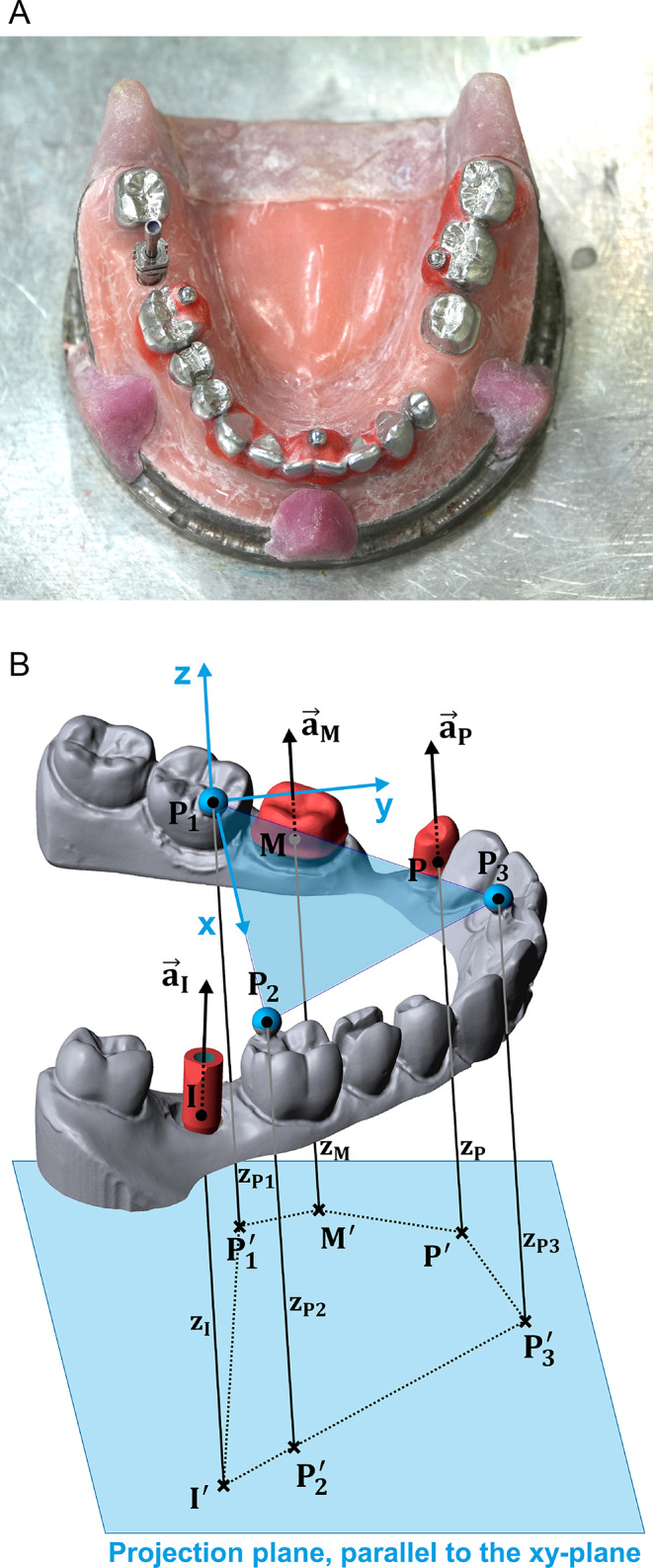
Metallic reference model of a lower jaw with prepared teeth in positions 36 and 34, an implant with scan body in position 47, and precision balls in regions 37, 11/12, and 46. Rest positions made of composite resin ensured a defined and position of the used impression trays (**a**). Precision ball center points ($\:{\text{P}}_{1}, {\text{P}}_{2}, {\text{P}}_{3}$) were used to define a global coordinate system as well as the horizontal plane. Reference points were also attached to the finishing line centers of teeth and scan abutment in regions 36 (M), 34 (P), and 47 (I). For separate evaluation of horizontal and vertical accuracy, all points were projected in a horizontally oriented plane (**b**)

Trays of the FDM and DLP group were designed with a commercial CAD Software (Dental Designer 2020, 3Shape, Copenhagen, Denmark). During the design process, custom tray wall thickness as well as spacing for the impression material were set to 3 mm which was the maximum wall thickness in the design process. It was ensured that the designed custom trays were in contact with the rest positions at the reference model enabling a reproducible tray positioning during impression making (Fig. 2a). The resulting spacing for the impression material was 3–4 mm. Additional construction of a borderlock and wall perforations were performed to gain additional retention of the impression material within the tray and thus to avoid detachment of the impression material from the tray during impression removal from the model. The final tray design was exported as a stl-file which could be used in the 3D-printing process. For both, FDM and DLP, printing settings as well as post-processing were chosen as specified by the manufacturers and described above.

For the fabrication of the MPR trays, the reference model was blocked out with a double layer of modeling wax (resulting thickness about 1.5–2 mm) heated in a water bath. Afterwards the reference model with the wax was digitalized (D2000; 3Shape) and the blocked-out model reproduced with an FDM printer (Raise3D Pro2) using the material Arfona Tray. This blocked-out model was then used as working base for all MPR trays. Before each tray was made from one plate of composite resin, this printed model was blocked out again with a double layer of modelling wax ensuring a realistic fabrication process and an easy separation of tray and printed model. At the end manually modelled resin trays were light-cured as given above. Retentive openings were added manually analogous to the digital design.

**Fig. 3:**
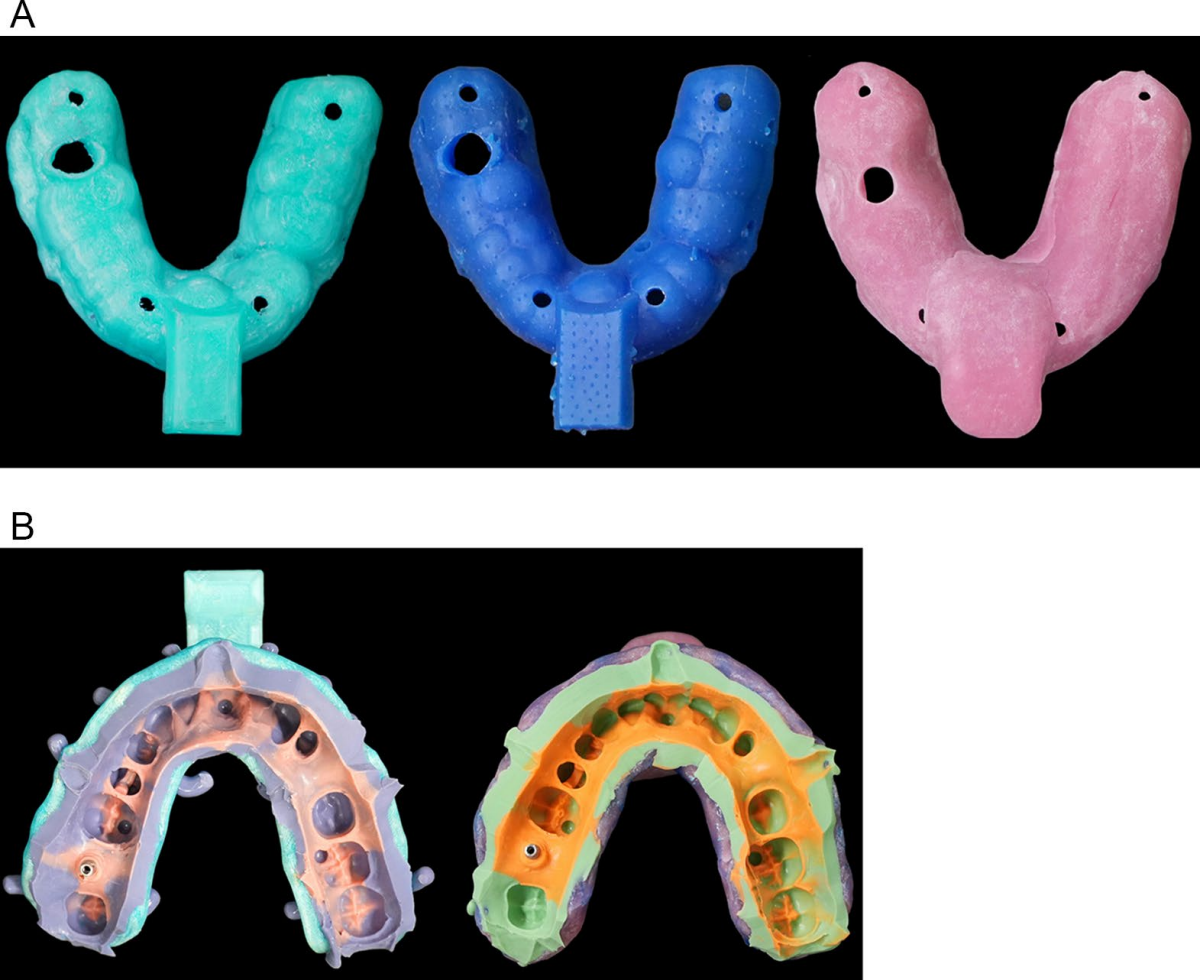
Impression trays either fabricated by 3D-printing or manual processing. From left to right: FDM impression tray, DLP impression tray, and MPR impression tray (**a**). Exemplary impressions (left: FDM impression tray, PE impression material, right: MPR impression tray, VPS impression material) containing the scan post (**b**)

Exemplary trays of all test groups are displayed in Fig. 3a. Before impression taking, trays were placed on the reference model with impression post (Fig. 2a) to check the correct position. Manual enlargement of the hole for the post was performed by the executing dentist (D.D.) if necessary.

#### Impression making and disinfection

Impressions (*n* = 10 / subgroup) were made using each combinations of VPS / PE and the tray materials Arfona Tray (group FDM), V-Print Tray (group DLP) and hand-made resin trays from LC Tray (group MPR). Inner surface of trays was brushed with the respective tray adhesive (PE: Impregum Penta H Duo Soft, LOT 9921089 / Impregum Garant L Duo Soft, LOT 9069392, 3 M Oralcare, VPS: Aquasil Ultra+ Heavy Regular Set, LOT 00065848 / Aquasil Ultra+ XLV Regular Set, LOT 00064055, Dentsply Sirona) which was gently dried with pressured oil-free air. Impression materials were used according to the manufacturers’ recommendations but the residence time of impressions on the model was doubled. All impression taken were stored for 5 min in a disinfection solution (Printosept ID, Alpro, Germany) and a minimum reset time of 60 min was awaited before fabrication of stone models. Same adhesives and material combinations were used for this main study as described in Chap. 2.1.

#### Fabrication and digitization of the stone models

Impressions were poured using class IV stone (Esthetic Base Gold, Dentona, Dortmund, Germany, LOT 2091030). Saw-cut models were fabricated using Giroform base plates (Amann Girrbach, Pforzheim, Germany). A magnetic split cast base plate of Giroform system was also permanently attached to the interface plate of the scanner to achieve a standardized model positioning during 3D scanning (D2000, 3shape). Scans were each were done according to the following regime: each twelve circumferentially distributed scans with tilts of 20° and 50° and 4 circumferentially distributed scans with 75° tilt with respect to the horizontal plane were performed with a resolution on 70 μm (Convince 2015, 3shape) and a rather fine and homogeneous mesh generated with a triangle edge length of 60 μm.

#### Accuracy measurements between the reference and stone models

**Fig. 4:**
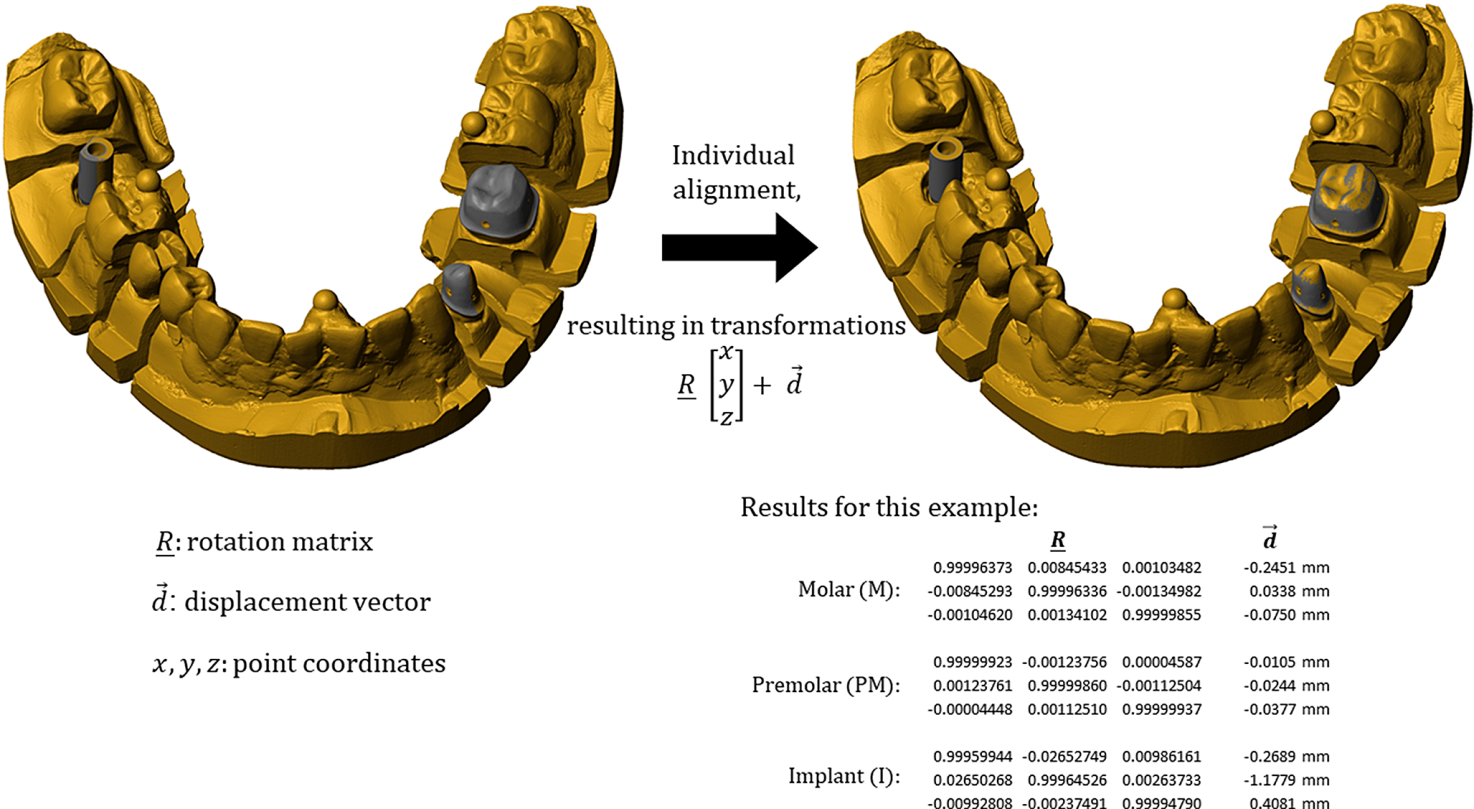
Model scan (beige) after alignment of scan coordinate system and global coordinate system of the reference model (grey) of which only the prepared teeth and scan body are displayed in this image (**a**). Reference teeth and scan body after individual alignment with the model scan and corresponding mathematical transformations (**b**)

Deviations between the digitized master model and the stone model scans were analyzed with the aid of Geomagic Design X V2022 (3D Systems, Rock Hill, SC, USA) and Matlab R2022a (Mathworks, Natick, MA, USA). For each scan, regions showing the precision balls were cropped and sphere center positions calculated by means of optimization (least squares, fixed nominal sphere radius). First, a coordinate transformation was carried out for the scan data aligning the scan coordinate system (given by the precision ball center points, same definition as given above for the reference data set) with the coordinate system of the reference model. In this state, surfaces of prepared teeth and scan body deviate between scan and reference (Fig. 4, left). To identify spatial position and orientation of each of the three reference surfaces was separately aligned with the respective surface region of the scan. The respective rotation matrix $\:\underline{R}$ contains the unit vectors of the tooth or scan body axes in the respective scan. With the known transformations (Fig. 4, right), points $\:{\text{M}},{\text{P}}$ and $\:{\text{I}}$ were taken along during these individual alignments (best fit, all constraints and limitations turned off). Thus, precision ball center points as well as position and orientation of the abutment teeth and scan abutment could be defined and projection of the points into the horizontal measurement plane carried out. Two different evaluations were conducted:

1. Global accuracy. Comparison of measures gained from the scans with the reference measures determined for the reference model:
  - Horizontal distance changes between all reference points (precision balls, abutment teeth, and scan abutment).
  - Angular changes in the horizontal plane between each three reference points.
  - Vertical distance changes between all reference points.
  - Angular changes between tooth and implant axes.
2. Local accuracy. After individual alignment of abutment teeth and scan abutment, distance deviations between reference and scan surfaces were calculated.
  - Trueness (mean deviation along each surface) and precision (standard deviation of measured distances along each surface) were given based on absolute deviation values.

### Sample size and statistical evaluation

All statistical tests were performed using SPSS Ver. 27 (IBM Corp., New York, NY, USA) at a significance level of α = 0.05.

Pre-tests on material parameters from bending tests (Young´s moduli und bending strengths) of the tray materials were not used for statistical testing but to help to interpretate the results of this investigation. The chosen sample size of *n* = 10 was appropriate to calculate mean values and standard deviations (SD) for each subgroup.

Peel bond strengths were statistically analyzed using means and SD, inter-group comparisons were performed using t-tests. In addition, results were visualized by use of boxplot diagrams. Since similar statistical effects were expected as published by Xu et al. [26], the same sample size (*n* = 12) was used for the peel bond tests in this investigation.

The main study on impression accuracy of individual trays was similar to an already published investigation on the impression accuracy of disposable custom plastic trays [23]. Since significant group differences could be identified in the previous publication also using PE and VPS impression materials with *n* = 10 / group, the identical sample size was used for the current study as well. Results were analyzed using mean values and SD and boxplot diagrams to visualize results where applicable. ANOVAs supplemented by post-hoc Tukey tests were compiled to test on possible influence of tray and impression materials and 2-sided inter-group comparisons, respectively, on accuracy parameters (global accuracy, trueness and precision). Boxplot diagrams were used where applicable for visualization.

## Results

### Material parameters

Young´s moduli (mean value ± SD) at room temperature / body temperature were 7.59 ± 0.54 GPa / 5.25 ± 0.73 GPa for MPR, 1.35 ± 0.05 GPa / 1.24 ± 0.07 GPa for FDM, and 2.41 ± 0.15 GPa / 1.87 ± 0.07 GPa for DLP. Young´s modulus of the plate material for manual processing was therefore about 3–5 times higher than measured for the 3D-printed materials. With regard to the trays fabricated from these materials, the wall thickness b is also of importance: bending stiffness ~ b^3^. With a mean plate thickness of b = 2.38 mm for MPR and b = 3.00 mm chosen for FDM and DLP trays, bending stiffness ratios for the tray walls are therefore by factor (3.00 / 2.38)^3^ = 2.003 smaller than the ratios given for the Young´s moduli.

Bending strengths at room temperature / body temperature were 79.1 MPa / 60.0 MPa for MPR, 59.1 MPa / 49.1 MPa for FDM, and 94.5 MPa / 64.2 MPa for DLP.

### Peel bond strength

**Fig. 5:**
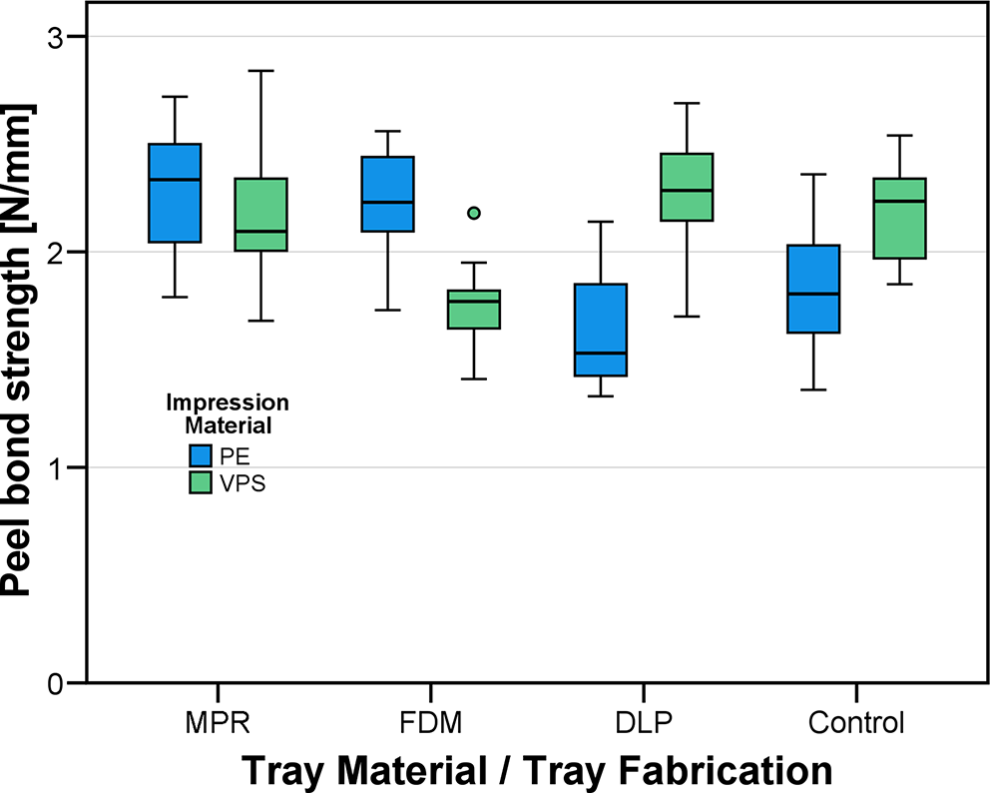
Measured peel bond strengths between combinations of impression (PE: polyether, VPS. vinylpolysiloxane) and tray materials (FDM: filament deposition modeling, DLP: digital light processing, MPR: manual processing, Control: stainless steel

Mean peel bond strengths ranged in 1.7 N/mm to 2.3 N/mm. Lowest peel bond strength values were given for combinations PE/DLP and VPS/FDM. All other test groups performed better than the respective control group with stainless steel as tray material. See Fig. 5 for visualization.

ANOVA revealed that selection of tray material had a significant impact on peel bond strength (*p* = 0.003), whilst impression material had not (*p* = 0.120). The factor interaction tray material * impression material was significant (*p* < 0.001). This was because PE performed better than VPS with MPR and FDM, but worse with than VPS with DLP and stainless steel (Control). With PE, peel bond strength of the control was slightly below 2 N/mm. Compared to this control group, group DLP showed similar values and groups MPR and FDM performed significantly better (*p* = 0.002 and *p* = 0.007, respectively). With VPS, the peel bond strength of the control group was slightly above 2 N/mm. Here, groups MPR and DLP performed significantly better than the control group while group FDM had significantly inferior peel bond strength (*p* = 0.001).

Significant group differences were found between FDM and MPR (*p* = 0.014), DLP and MPR (*p* = 0.004) and MPR and steel (Control group, *p* = 0.031). All other comparisons missed the level of statistical significance (*p* > 0.05).

### Impression accuracy

#### Global accuracy: distance, angular, and axis deviations

**Fig. 6:**
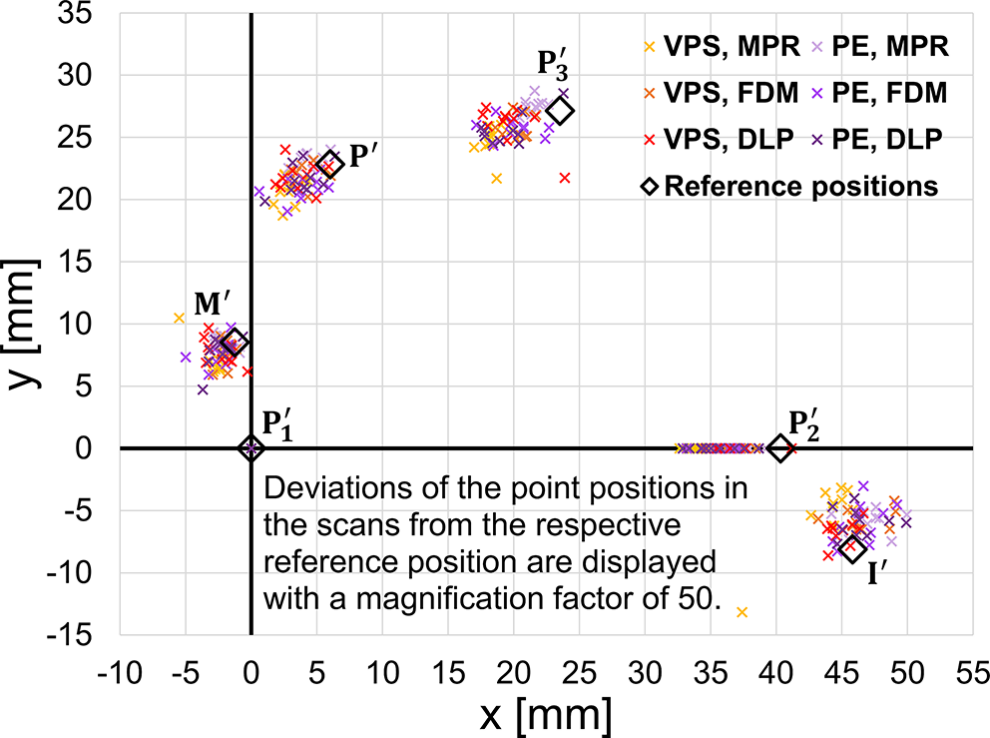
Point positions in the horizontal plane calculated for all model scans of the test groups differing in impression (PE: polyether, VPS. vinylpolysiloxane) and tray material (FDM: filament deposition modelling, DLP: digital light processing, MPR: manual processing). Distance deviations from the reference positions are displayed with a magnification factor of 50

In Fig. 6, the distribution of the calculated precision ball center points ($\:{\text{P}}_{1}^{{\prime\:}}$_,_$\:{\text{P}}_{2}^{{\prime\:}},{\text{P}}_{3}^{{\prime\:}}$) and finishing line center points ($\:{M}^{{\prime\:}},{P}^{{\prime\:}},{I}^{{\prime\:}}$) projected in a horizontal plane is depicted. Due to coordinate system alignment, $\:{\text{P}}_{1}$ is identical with the origin in each scan and $\:{\text{P}}_{2}$ is located on the x-axis. With the exception of few scans, all test data showed the same displacement pattern: The geometry given by the stone cast scans tended to be smaller than that of the reference model. However, the displacements found for the Implant ($\:{I}^{{\prime\:}}$) were much smaller compared to displacements of the other elements in the second quadrant ($\:{\text{P}}_{2}^{{\prime\:}},{\text{P}}_{3}^{{\prime\:}}$).

**Fig. 7:**
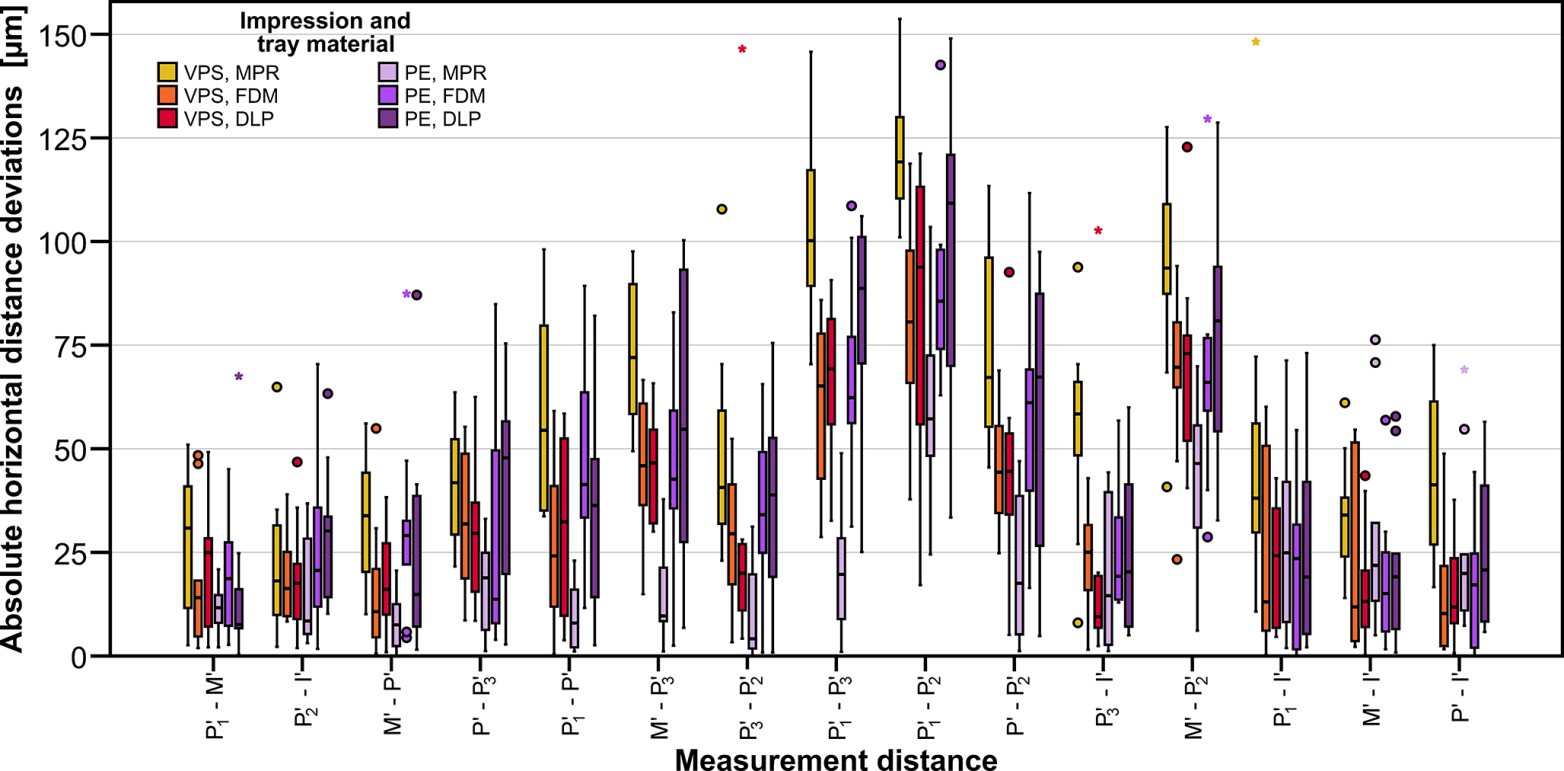
Boxplot diagram showing absolute horizontal deviations for the test groups differing in impression (PE: polyether, VPS. vinylpolysiloxane) and tray material (FDM: filament deposition modeling, DLP: digital light processing, MPR: manual processing). Measurement distances between pairs of reference points are sorted in ascending order

The absolute horizontal distance deviations (Fig. 7) were significantly affected by the impression material (*p* < 0.001) whereas the tray material (*p* = 0.192) had no significant impact on the results. However, the factor combination impression material * tray material was highly significant (*p* < 0.001). This finding makes sense when taking into account that for impression material VPS tray materials FDM and DLP performed significantly better than MPR whereas with impression material PE tray material MPR led to significantly smaller deviations compared to FDM and DLP. The factor measurement distance also had a significant influence on the measured horizontal distance deviations (*p* < 0.001), i.e., longer distances correlated in general with larger deviations. The exceptions, as already seen above, were all distances including the implant. Highest deviations were given for the distance $\:{\text{P}}_{1}^{{\prime\:}}-{\text{P}}_{2}^{{\prime\:}}$ (mean deviation over all test groups: 89 μm). Each significantly lower deviations were recorded for subsets [$\:{M}^{{\prime\:}}-{\text{P}}_{2}^{{\prime\:}}$ (70 μm) and $\:{\text{P}}_{1}^{{\prime\:}}-{\text{P}}_{3}^{{\prime\:}}$ (66 μm)] and [$\:{P}^{{\prime\:}}-{\text{P}}_{2}^{{\prime\:}}$ (51 μm) and $\:{M}^{{\prime\:}}-{\text{P}}_{3}^{{\prime\:}}$ (47 μm)]. Mean deviations for all other measurement distances, including all pairs with the implant, were ≤ 35 μm. The significantly smallest distance deviation was given for $\:{{\text{P}}_{1}^{{\prime\:}}-M}^{{\prime\:}}$ (19 μm) and $\:{{\text{P}}_{2}^{{\prime\:}}-I}^{{\prime\:}}$ (22 μm). Angular changes in the horizontal plane were similar for all test groups with mean angular deviations in each test group of about 0.1° and maximum angular deviations below 0.9°.

**Fig. 8:**
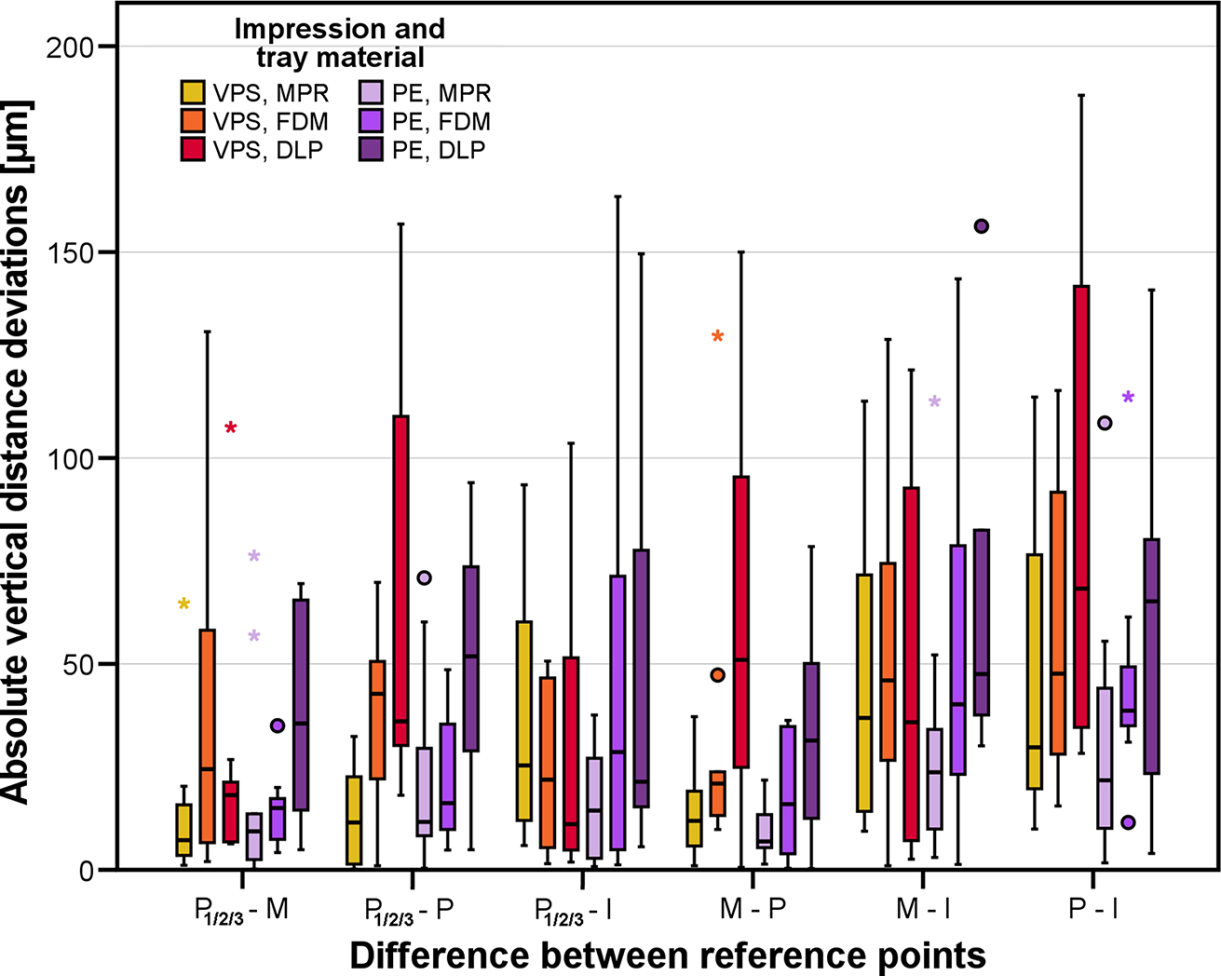
Boxplot diagram showing absolute vertical deviations for the test groups differing in impression (PE: polyether, VPS. vinylpolysiloxane) and tray material (FDM: filament deposition modeling, DLP: digital light processing, MPR: manual processing)

The absolute vertical distance deviation can be seen in Fig. 8. ANOVA revealed a different behavior than found for the horizontal distance deviations: Here, the impression material had no significant effect (*p* = 0.308) while significantly different results were identified between all groups differing in tray material (*p* < 0.001): Mean vertical differences were lowest with tray material MPR (18 μm) followed by FDM (27 μm) and finally DLP (38 μm). The influence of the factor combination impression material * tray material was not significant (*p* = 0.232). It should be noted that maximum vertical errors recorded for all test groups differing in tray and impression material ranged between 100 μm and 150 μm.

**Fig. 9:**
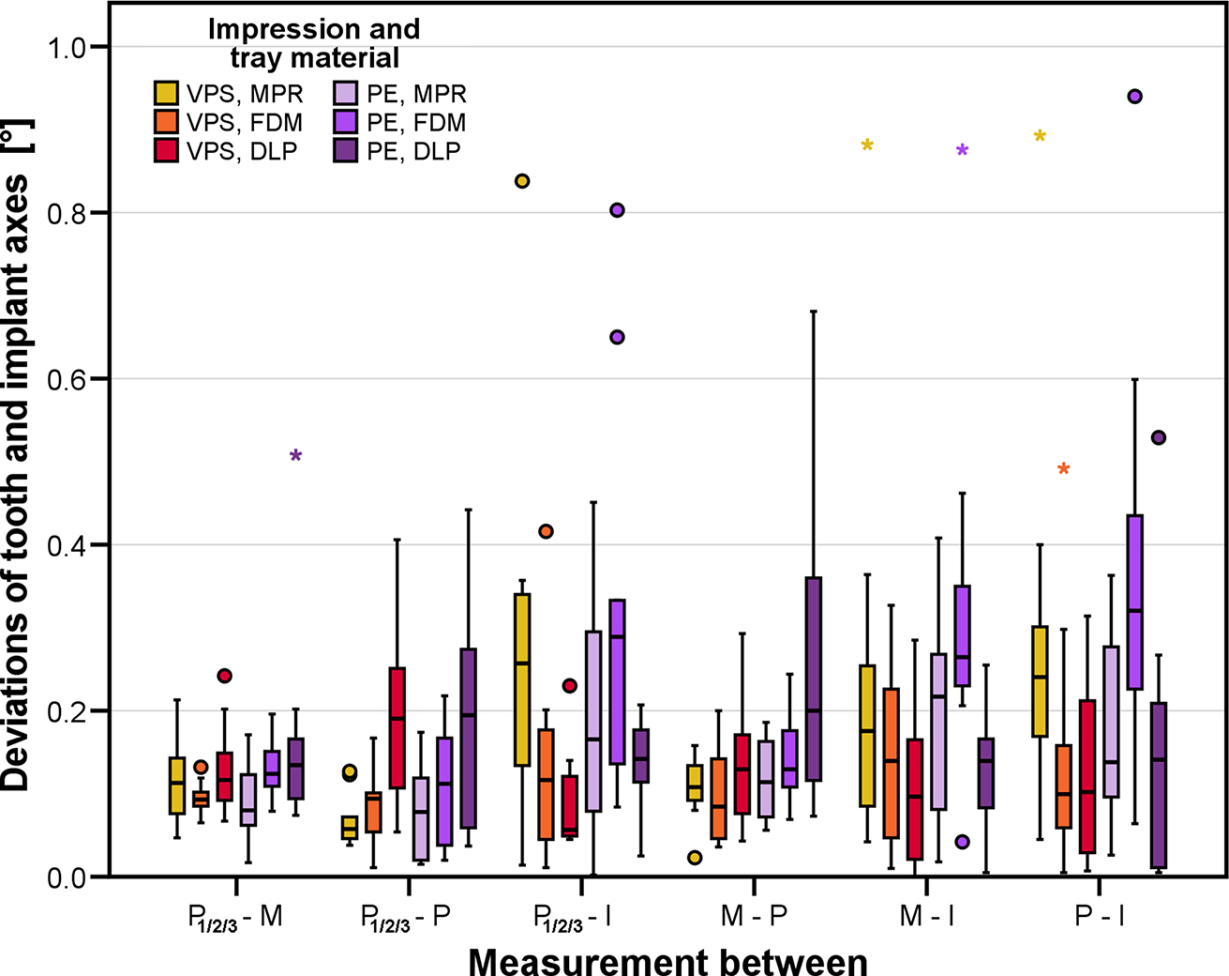
Deviations between the axes pairs of prepared teeth, implant as well as the horizontal plane P_1/2/3_ for the test groups differing in impression (PE: polyether, VPS. vinylpolysiloxane) and tray material (FDM: filament deposition modeling, DLP: digital light processing, MPR: manual processing)

For the angular changes between tooth and implant axes (Fig. 9), only impression material (*p* = 0.001, mean angular changes 0.11° with PVS and 0.14° with PE) and the combination of impression material and tray material had a significant influence. Again, the difference between the two impression materials was clinically not relevant, but the combinations of impression material and tray material should be regarded more closely: With VPS impressions, best results were given for FDM tray whereas PE impressions performed best with MPR.

#### Local accuracy: surface deviations of the prepared teeth

Local accuracy given by trueness and precision based on individual absolute deviations along the abutment surfaces was excellent for all test groups. Detailed trueness and precision results for the prepared premolar and molar as well as the scan abutment placed on the implant are presented in Table 2. Trueness was significantly affected by all factors (impression material, tray material, abutment, *p* ≤ 0.011) while significant influence on precision was only given for factors tray material and abutment (*p* ≤ 0.005) and not the factor impression material (*p* = 0.682). Best results were given for combinations VPS/MPR and PE/FDM. Regarding the abutments, trueness of the prepared molar (overall mean value 5.6 μm) was significantly better than found for both, prepared premolar (8.2 μm) and scan abutment (8.1 μm). Similar results were given for precision where results of all abutments differed significantly (molar: 4.4 μm, premolar: 5.3 μm, and scan abutment 7.5 μm). However, differences in mean values for trueness and precision for the test groups were small and clinically not relevant.

**Table 2:** Results for the local accuracy measured after individual alignment of the prepared teeth and the scan abutment given for the test groups differing in impression (PE: polyether, VPS. Vinylpolysiloxane) and tray material (FDM: filament deposition modeling, DLP: digital light processing, MPR: manual processing)

Tray / Impression material	Abutment	Trueness [µm]	Precision [µm]
VPS / MPR	Molar	5.1 ± 0.8	4.4 ± 0.7
	Premolar	5.8 ± 1.4	4.5 ± 0.7
	Implant	7.7 ± 0.5	7.5 ± 0.2
	All abutments	6.2 ± 1.5	5.5 ± 1.5
VPS / FDM	Molar	5.3 ± 1.9	4.0 ± 1.0
	Premolar	8.2 ± 3.5	5.2 ± 1.5
	Implant	9.5 ± 5.3	7.4 ± 0.4
	All abutments	7.7 ± 4.1	5.5 ± 1.8
VPS / DLP	Molar	7.3 ± 2.7	5.3 ± 1.9
	Premolar	9.9 ± 5.1	6.2 ± 2.5
	Implant	11.3 ± 4.5	7.6 ± 0.7
	All abutments	9.5 ± 4.4	6.4 ± 2.1
PE / MPR	Molar	6.2 ± 0.9	5.0 ± 0.7
	Premolar	9.1 ± 2.6	5.9 ± 0.9
	Implant	7.1 ± 0.5	7.5 ± 0.4
	All abutments	7.5 ± 2.0	6.1 ± 1.3
PE / FDM	Molar	4.4 ± 0.5	3.6 ± 0.4
	Premolar	6.7 ± 1.5	4.6 ± 0.7
	Implant	6.4 ± 0.5	7.5 ± 0.4
	All abutments	5.8 ± 1.4	5.2 ± 1.7
PE / DLP	Molar	5.3 ± 2.1	4.2 ± 1.1
	Premolar	9.5 ± 2.4	5.8 ± 1.0
	Implant	6.7 ± 0.4	7.4 ± 0.3
	All abutments	7.2 ± 2.5	5.8 ± 1.6

## Discussion

The study hypotheses had to be partly rejected. Global accuracy was significantly influenced by measurement paths’ lengths and tray / impression material selection, suggesting rejection of the null hypothesis, albeit partially clinically of minor relevance. Trueness was affected by the abutment studied and tray and impression material whilst precision was only crossed with the variables abutment and tray material. Regarding the global accuracy (distance deviations and angular deviations) longer measurement paths led to less accuracy. Interestingly, measurements which included the implant situation rather tended to be more accurate replications of the model situation. One might argue that the use of a transfer abutment for impression making leads to less distortions of the impression material while demolding in comparison to impression areas with prepared tooth structures. Whereas the removal of the impression post from the implant body after screw loosening can be done without exertion of tensile forces, such forces are exerted on unprepared and prepared teeth to overcome retention during removal of the impression. In this context, retention forces with PE impressions are about twice as high as those occurring with VPS impressions [23]. It is also expected from previous studies that longer distances rather led to greater distance deviations [9, 16, 23, 27]. Nearly ubiquitously, measurement paths in comparison of replica and reference models were in mean shorter which can be justified by shrinkage of the impression material and is in accordance to previous studies [9, 23, 28]. Although the measured peel bond strength values were appropriate, additional mechanical retention elements in form of perforations (Fig. 3) were added to the individual tray design to further minimize the risk of delaminations between tray and impression material. In contrast to a previous study with custom plastic tray and models made of zero-expansion stone, an expansion < 0.08% was given by the manufacturer for the type 4 stone used in this study. With the use of saw-cut models, however, (theoretical) changes in measured distances due type 4 stone expansion values are clearly below 10 μm. When looking at the of single prepared teeth or the implant scan body, their surface geometries were given with high local accuracy: Mean trueness and precision were smaller than 9 μm. Thus, accurate geometry assessment of single elements does, in general, not state a problem but the relative spatial relation of each two elements, the global accuracy, was associated with larger distance deviations. However, all tray materials and impressions materials yielded mean inaccuracies far below 100 μm which probably would lead to acceptable fitting fixed dental prostheses during prosthetic treatment. Therefore, the material and tray combinations can be recommended for further clinical investigations.

Overall best global accuracy results were correlated with MPR trays, probably due to their high stiffness. An influence of the trays´ bending stiffnesses was expected by the authors in advance of the investigation. This was the reason for choosing a higher wall thickness for 3D-printed trays made of resin (FDM and DLP, 3.0 mm wall thickness) compared manually fabricated trays made of composite (MPR, 2.4 mm wall thickness). Even with that adaptation, MPR trays had about twice the bending stiffness compared to 3D-printed trays. In consequence, especially with PE impressions needing high removal forces, deformations and distortions of the tray (as well as the included impression material) were much smaller in the MPR group compared to DLP and FDM test groups. It can be speculated that this behavior was one key factor resulting in the findings displayed in Figs. 6 and 7. Only for horizontal distance deviations with VPS impression did 3D-printed trays (FDM, DLP) outperform MPR trays. Up to date 3D-printable tray materials are pure resins. It would be interesting to print trays from resin materials to check if some of the identified drawbacks can be eliminated when using a material with higher Young´s modulus. Literature on the accuracy of impression performed with 3D printed trays is scares and inhomogenous in indications and outcome. A previous study found that 3D printed trays lead to a higher impression accuracy compared to individual hand-made resin trays if implants were the target abutment [21]. Another study concluded similarly when impressions of the edentulous jaws were made. Contrary, another author group found that hand-made trays yield the best possible accuracy [20]. No evidence is available on filament printed tray materials. Those materials are especially interesting as the can be fitted with favorable properties like biodegradability.

When interpreting and transferring the results of this study into clinic, one should keep in mind that this is an in vitro investigation and thus extrapolation of the outcome should be done with caution. Albeit several standardizations such as controlled equal positioning of the trays, exact material use according to manufactures’ recommendations and precise scanning approaches – amongst a variety of other attempts to keep conditions equally - were applied in this study, accuracy is affected by bleeding of abutment teeth, humidity of the oral cavity, body temperature, subgingival preparation margins and gingiva retraction. Here, the different materials used in this study may also behave differently among each other. On the other hand, for specific material combinations the laboratory and clinical process chain (i.e. shrinkage of impressions, expansion of stone cast) can lead to a compensation of inaccuracies as seen in vitro. However, the study results can paint a picture of fundamental material properties and resulting accuracy. The use of very precise scanning devices and a dedicated typodont model enables to possibility to assess the accuracy in a validated manner. To the knowledge of the authors this is the first study on accuracy of filament printed trays in use with VPS and PE impressions and the outcome can therefore be the basis for clinical studies.

## Conclusions

The observed distortions were small for impressions taken by use of polyether (Impregum) and vinylpolysiloxane (Aquasil Ultra+) using different 3D-printed (Arfona Tray filament and V-Print Tray resin) and hand-made trays. All material combinations studied presented an adequate level of accuracy making them recommendable at least for use of tooth / implant supported single crowns and small bridges. For restorations requiring utmost accuracy, manually manufactured individual impressions trays should be preferred. Clinical studies should prove the laboratory outcome.

## Data Availability

No datasets were generated or analysed during the current study.
